# A low albumin level as a risk factor for transient severe motion artifact induced by gadoxetate disodium administration: A retrospective observational study with free-breathing dynamic MRI and an experimental study in rats

**DOI:** 10.1371/journal.pone.0265588

**Published:** 2022-03-18

**Authors:** Takumi Sugiura, Kenichiro Okumura, Motomitsu Sasaki, Junichi Matsumoto, Takahiro Ogi, Norihide Yoneda, Azusa Kitao, Kazuto Kozaka, Wataru Koda, Satoshi Kobayashi, Toshifumi Gabata

**Affiliations:** 1 Department of Radiology, Kanazawa University Graduate School of Medical Sciences, Kanazawa, Ishikawa, Japan; 2 Department of Radiology, Nakamura Hospital, Echizen, Fukui, Japan; 3 Department of Quantum Medical Technology, Kanazawa University Graduate School of Medical Sciences, Kanazawa, Ishikawa, Japan; Humanitas Clinical and Research Center - IRRCS, ITALY

## Abstract

**Objectives:**

In the arterial phase of gadoxetate disodium administration for dynamic MRI, transient severe motion (TSM) sometimes occurs, making image evaluation difficult. This study was to identify risk factors for TSM in a clinical study, and confirm them and investigate the cause in an animal study.

**Methods:**

A retrospective, single-center, observational study included patients who underwent dynamic MRI using gadoxetate disodium for the first time from April 2016 to September 2019 and free-breathing MRI was performed. Differences in clinical characteristics and laboratory tests between the presence and absence of TSM were examined. Animal experiments were conducted in 50 rats; gadoxetate disodium was injected into three sites (distal inferior vena cava (IVC), ascending aorta, and descending aorta) to identify the organ which triggers respiratory irregularities. Phosphate-buffered saline and gadopentetate dimeglumine were also injected into the distal IVC. In addition, to evaluate the effect of albumin, gadoxetate disodium was diluted with phosphate-buffered saline or 5% human serum albumin and injected into the ascending aorta. The time course of the respiratory rate was monitored and evaluated.

**Results:**

20 of 51 (39.2%) patients showed TSM. On multivariable analysis, a low albumin level was an independent risk factor (*P* = .035). Gadoxetate disodium administration caused significant tachypnea compared to gadopentetate dimeglumine or PBS (an elevation of 16.6 vs 3.0 or 4.3 breaths/min; both *P* < .001) in rats. The starting time of tachypnea was earlier with injection into the ascending aorta than into the descending aorta (10.3 vs 17.9 sec; *P* < .001) and the distal IVC (vs 15.6 sec; *P* < .001). With dilution with albumin instead of phosphate-buffered saline, tachypnea was delayed and suppressed (9.9 vs 13.0 sec; *P* < .001, 24.1 vs 17.0 breaths/min; *P* = .031).

**Conclusions:**

A low albumin level is a risk factor for TSM, which could be caused by the effect of gadoxetate disodium on the head and neck region.

## Introduction

Gadoxetate disodium is a paramagnetic, gadolinium-containing, hepatocyte-specific contrast agent widely used in hepatobiliary diagnostic imaging because of its unique pharmacokinetic profile and its ability to be rapidly taken up by hepatocytes [1–7].

However, in recent years, it has been reported that, in the acute phase of gadolinium-containing contrast agent administration, especially with gadoxetate disodium, transient severe motion (TSM) that does not depend on dyspnea occurs, and it is often difficult to evaluate the arterial phase image during dynamic imaging [8–11]. Even under free breathing, i.e. without dyspnea caused by breath holding, a prospective observational cohort study showed that gadoxetate disodium administration can lead to TSM [12].

To the best of our knowledge, the causes of gadoxetate disodium-induced TSM have not been adequately studied [10, 12, 13]. The purposes of this study were to identify risk factors for the occurrence of TSM in a clinical retrospective analysis and to confirm them and investigate the cause in animal experiments.

## Materials and methods

This study consisted of a clinical study and an animal study.

### Clinical study

A retrospective, single-center, observational study was conducted in accordance with the ethical standards of the Declaration of Helsinki and was approved by our institutional review board (registration number 2019001). Because of the retrospective nature of this study, a waiver of written informed consent was approved.

#### Study population

Consecutive patients who underwent abdominal multiphase, contrast-enhanced MRI using gadoxetate disodium for the first time during the period from April 2016 to September 2019 for detection of metastatic liver tumor or hepatocellular carcinoma were included and free-breathing MRI was performed to avoid the effect of breath-holding fidelity.

#### MRI parameters and contrast agent protocol

MRI was performed using a 1.5-T system (Optima MR360 Advance; GE Healthcare, Chicago, IL, USA) with a 12-channel phased-array coil using a free-breathing approach. Dynamic contrast-enhanced T1-weighted imaging was performed using three-dimensional fat-suppressed fast spoiled gradient-echo acquisition with a respiratory trigger with true in-plane spatial resolution of 1.25 × 2.25 × 5 mm; flip angle, 15°; repetition time (msec)/echo time (msec), 3.8/1.4; section thickness, 5mm; field of view, 360mm; 288 × 160 matrix. Image acquisition time was approximately 22 sec. Gadoxetate disodium (Primovist, Bayer Yakuhin, Osaka, Japan) was administered intravenously at a standard dose of 0.025 mmol/kg body weight and a rate of 1 or 2 mL/sec, followed by a saline flush at an equivalent rate.

#### Assessment of images and respiratory irregularities

**Fig 1:**
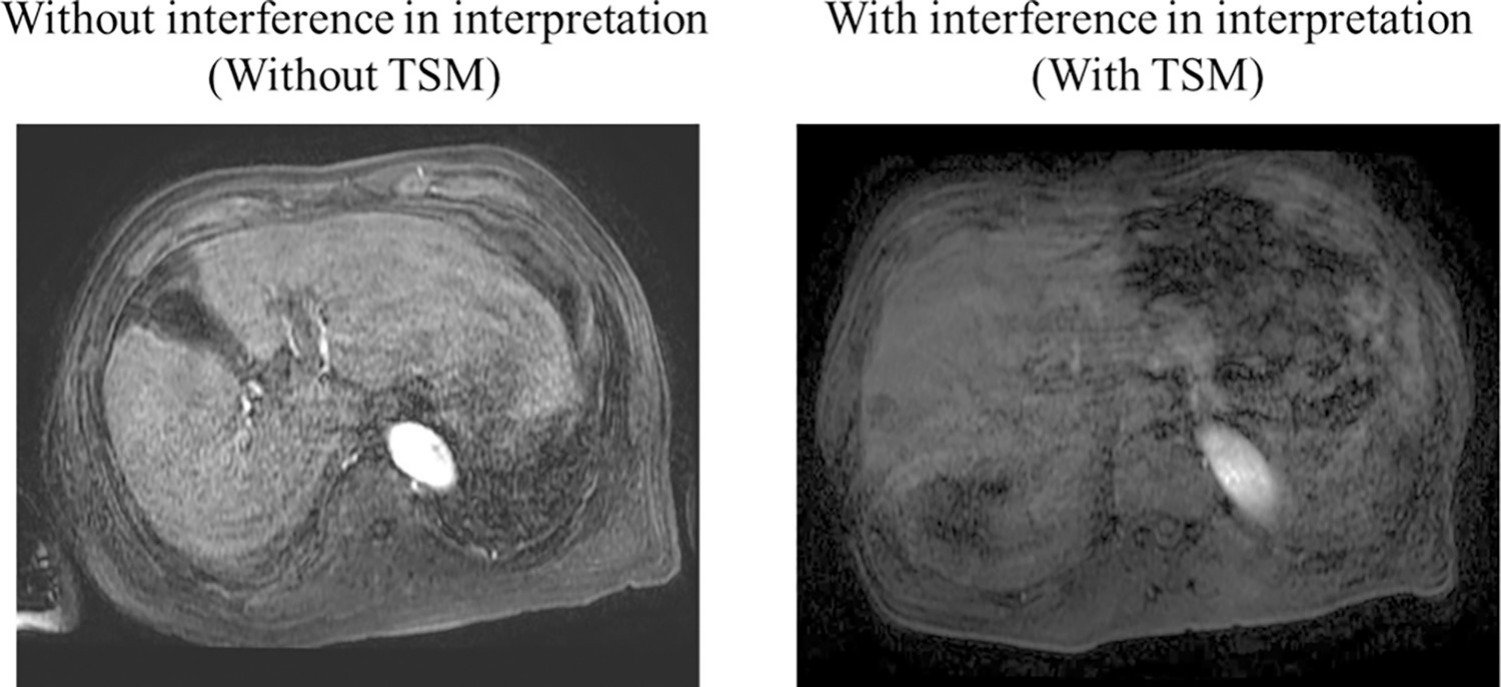
Representative examples of disturbances without or with interference in image interpretation (Without or with TSM). Note–TSM, transient severe motion.

Visual assessment of the pre-contrast and arterial phase images was performed. Two radiologists with 12 and 30 years of clinical experience in abdominal imaging (K.O. and S.K., respectively) evaluated the severity of motion-related artifacts by consensus and whether the artifact interfered with image interpretation (TSM; Fig 1). The reviewers assessed the image series in terms of the degree of image deterioration presumably caused by abdominal movement. Reviewers were specifically instructed to ignore other sources of artifacts, particularly truncation artifact. Reviewers were blinded to any data without images. The artifact score was defined as in the previous report [14]: score 1, no artifact; score 2, mild artifact, not interfering with diagnostic assessment; score 3, moderate artifact affecting diagnostic assessment; and score 4, severe artifact rendering image nondiagnostic. TSM that interferes with image interpretation includes scores 3 and 4.

### Animal study

Animal studies were conducted to evaluate the effect of gadoxetate disodium administration, and the differences in the respiratory rate (RR) among different injection sites of gadoxetate disodium (distal inferior vena cava (IVC), ascending aorta, and descending aorta) to identify the organ which triggers respiratory irregularities. The effect of serum albumin was also evaluated, which was identified as a risk factor for TSM in the clinical study reported in this article (for details, see Results below). All animal experiments were conducted in accordance with the guidelines of our institution and approved by our animal research committee (study protocol ID: AP194103).

#### Animals

**Fig 2:**
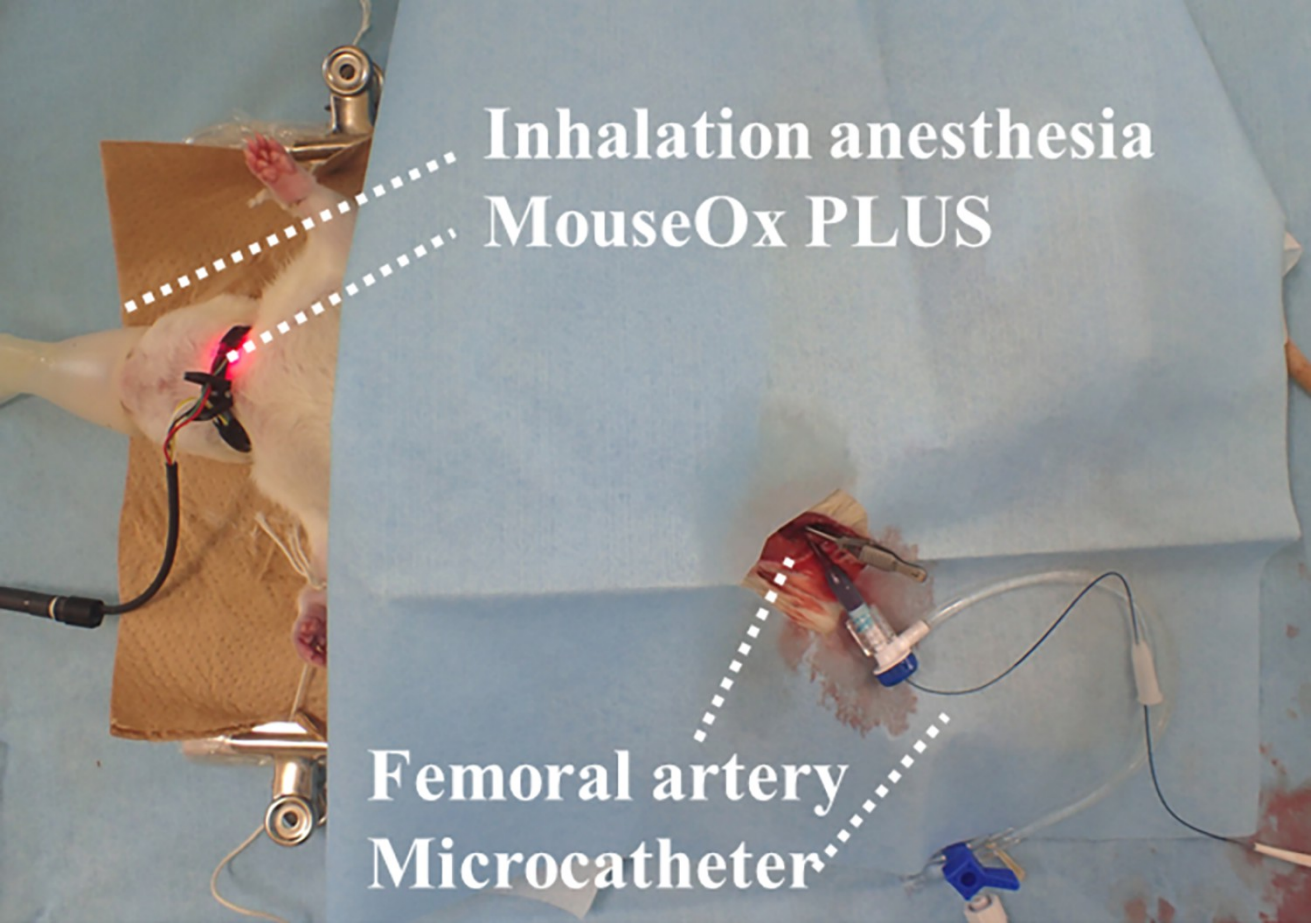
Representative rat experiment. Rats were anesthetized with 1.5% isoflurane in air. Vital signs including peripheral oxygen saturation (%), heart rate (beats/min), and respiratory rate (breaths/min) were monitored by MouseOx PLUS (Starr Life Sciences Corp., Oakmont, PA, USA). A microcatheter was inserted through the femoral artery.

Fifty male Sprague-Dawley rats purchased from Charles River Laboratories Japan were maintained in a specific pathogen-free facility. All rats weighed approximately 500 g and had free access to food and water. The rats were anesthetized with 1.5% isoflurane in air. The neck of each rat was shaved, the clip sensor was attached, and RR (breaths/min) was monitored every second and recorded by MouseOx PLUS (Starr Life Sciences Corp., Oakmont, PA, USA) (Fig 2). RR was shown as an average of 10 respiratory cycles.

#### Gadoxetate disodium administration and the differences by injection sites and digital subtraction angiography (DSA) with iodinated contrast agent

Angiography (Arcadis Avantic, Siemens, Munich, Germany) was performed by two radiologists (T.S. and K.O., with 8 and 12 years of clinical experience in interventional radiology, respectively). A 1.6-Fr microcatheter Carnelian MARVEL (Tokai Medical Products, Kasugai, Japan) was positioned at the distal IVC in 18 rats. Phosphate-buffered saline (PBS) alone and gadoxetate disodium diluted 4-fold with PBS were injected at a flow rate of 0.1mL/sec (total 1 mL) in 9 rats. In addition, gadopentetate dimeglumine diluted 2-fold with PBS was injected in 9 rats. A microcatheter was positioned at the ascending aorta or the descending aorta in 9 rats each, and PBS-diluted gadoxetate disodium was injected. Additionally, 1 mL of an iodinated contrast agent (Omnipaque 300 mgI/mL, GE Healthcare Japan, Hino, Japan) was injected into the distal IVC and the descending aorta at the same rate (0.1 mL/sec) for DSA.

#### Difference by dilution methods with 5% albumin or PBS

In addition, a microcatheter was positioned at the ascending aorta through the femoral artery in 14 rats. To assess the effect of albumin, gadoxetate disodium was diluted 4-fold with PBS or 5% human serum albumin (Albuminar-5; CSL Behring, Tokyo, Japan) injected at a flow rate of 0.1 mL/sec (total 1 mL). Each dilution was injected into 7 rats. Contrast media were injected into the ascending aorta because in the animal study of this article, the strongest respiratory irregularity was induced with injection into the ascending aorta (for details, see Results below).

#### Contrast media administration and RR monitoring

The rats were administered 0.12 mmol/kg gadoxetate disodium or 0.49 mmol/kg gadopentetate disodium in 1mL at a flow rate of 0.1 mL/sec with a commercially available injector (Model Fusion 710, Chemyx, Inc., Stafford, TX, USA). These doses are equivalent to the clinically approved human dose (human equivalent dose) after adjustment for body surface area, as recommended by the United States Food and Drug Administration. The experimenter was not blinded to the agents injected.

**Fig 3:**
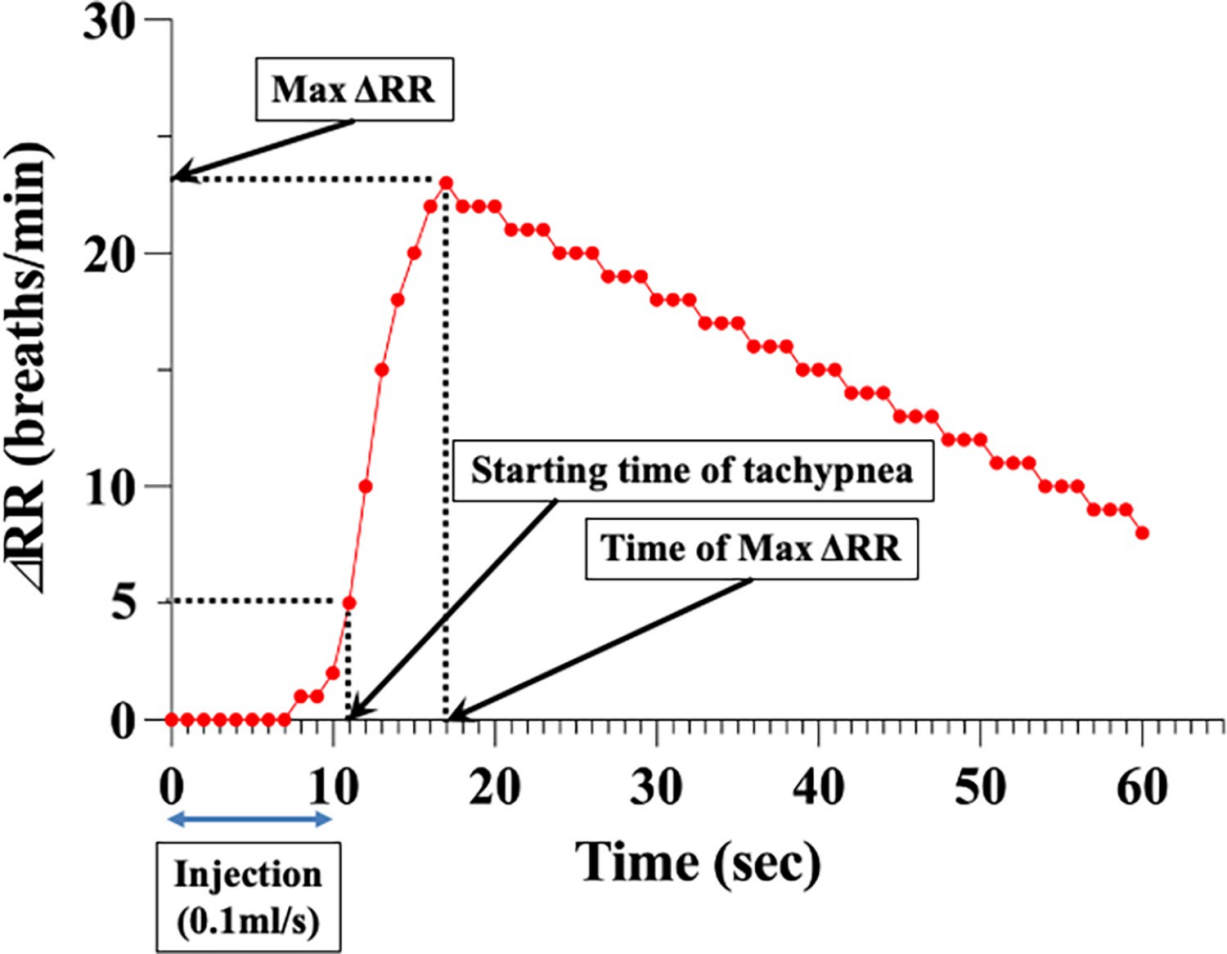
Definition of parameters related to the time course of the respiratory rate analysis. The starting time of tachypnea was defined as the time when the respiratory rate (RR) increased by more than 5 breaths/min compared to baseline (the time of the beginning of contrast medium injection). Max ΔRR was defined as the maximum value of ΔRR. Time of max ΔRR was defined as the time at which ΔRR reached its maximum value. In this manner, the starting time of tachypnea, max ΔRR and time of max ΔRR in individual rats are recorded and averaged for each experimental group. Note–ΔRR, change (increase) in the respiratory rate compared to baseline (0 sec; the time of the beginning of contrast medium injection). Starting time of tachypnea, the time when ΔRR reached 5 breaths/min.

Before injecting the test agents, RR was controlled to be in the range of 55 to 65 breaths/min as much as possible by changing the concentration of isoflurane. Steady state was confirmed for at least 10 min before injection. RR was recorded from 30 sec before until 60 sec after injection. The starting time of tachypnea was defined as the time point that showed an elevation in RR of more than 5 breaths/min compared to baseline (0 sec, Fig 3). The time and the value of maximum elevation were also evaluated.

### Statistical analysis

Values are shown as means (standard deviation). GraphPad (version 8.31 for Mac; GraphPad Software, San Diego, CA, USA) was used for statistical analysis. Descriptive statistics were examined to investigate the differences in clinical characteristics and the values of laboratory tests between the presence and absence of TSM by the unpaired *t*-test and the Mann-Whitney U test. The laboratory test analyses and clinical variables associated with TSM were entered into a logistic regression model to assess their relative contributions. Comparisons of time and elevation in RR among different materials, injection sites or dilution methods were also evaluated by one-way analysis of variance and Holm-Sidak’s multiple comparisons test or the unpaired *t*-test. A *P-*value < .05 was considered statistically significant.

## Results

### Results of the retrospective clinical study

#### Patients’ characteristics

The mean age and body mass index of the patients in this study were 71 ± 13 years (range, 44–91 years) and 21.9 ± 2.9 kg/m^2^ (range, 15.8–28.3 kg/m^2^), respectively. Overall, 31 of 51 (60.8%) patients were male, and 14 of 51 (27.5%) patients were current smokers, with lung disease in 6 of 51 (11.8%), chronic liver injury in 14 of 51 (27.5%), pleural effusion in 3 of 51 (5.9%), and ascites in 4 of 51 (7.8%). None of the patients had presented with neurologic symptoms that warranted head MRI.

#### Clinical characteristics of patients with TSM

Of the 51 patients, 20 (39.2%) showed TSM (Table 1). There were no significant differences in age, body mass index, smoking status, lung disease, chronic liver injury, pleural effusion, or respiratory irregularities between the two groups (*P* = .74, .16, .99, .90, .20, .11, and .15, respectively). Among clinical characteristics, a significant difference in sex (*P* = .049) was found, with male sex as a risk factor. On univariable analysis, no significant differences were found in white blood cells, hemoglobin, platelets, total bilirubin, or blood urea nitrogen between the two groups. On the other hand, hematocrit and creatinine were borderline significantly different (*P* = .066 and .061, respectively), and albumin was significantly different (*P* = .013).

**Table 1:** Comparisons of clinical characteristics and laboratory test results between patients with and without TSM.

	Artifacts without interference (Without TSM)	Artifacts with interference (With TSM)	*P value
	*n* = 31	*n* = 20	
Characteristic			
**Age, y, mean ± SD**	71 **±** 14	72 **±** 11	.74
**BMI, kg/m** ^**2**^ **, mean ± SD**	21.5 **±** 2.9	22.6 **±** 2.9	.16
**Sex**			
Male	15 (48.4)	16 (80)	**.049**
Female	16 (51.6)	4 (20)	
**Injection rate**			
1 mL/sec	23 (74.2)	15 (75)	.79
2 mL/sec	8 (25.8)	5 (25)	
**Current smoker**	8 (25.8)	6 (30)	> .99
**Lung disease (COPD or IP)**	3 (9.7)	3 (15)	.90
**Chronic liver injury (LC or CH)**	11 (35.5)	3 (15)	.20
**Pleural effusion**	0 (0)	3 (15)	.11
**Ascites**	2 (6.5)	2 (10)	.94
Laboratory test			
**WBC, ×10**^**2**^ **/μL**	62.5 (22.8)	60.1 (16.4)	.69
**Hb, g/dL**	12.3 (2.5)	11.3 (1.9)	.11
**Ht, %**	37.4 (6.4)	34.1 (5.7)	**.066**
**Plt, ×10** ^**4**^ **/μL**	23.0 (8.2)	25.5 (10.4)	.33
**Alb, g/dL**	3.7 (0.5)	3.3 (0.7)	**.013**
**T-Bil, mg/dL**	1.01 (0.6)	1.94 (3.9)	.65
**BUN, mg/dL**	14.0 (3.7)	15.8 (6.2)	.23
**Cr, mg/dL**	0.80 (0.19)	0.95 (0.3)	**.061**

Note.—Patient age and body mass index are given as means **±** standard deviation (SD). All other characteristics are summarized as raw data, with percentages of the overall population given in parentheses. The patient laboratory test data are reported as means (standard deviation).

TSM = Transient severe motion, BMI = body mass index, COPD = chronic obstructive pulmonary disease, IP = interstitial pneumonia, LC = liver cirrhosis, CH = chronic hepatitis, WBC = white blood cells, Hb = hemoglobin, Ht = hematocrit, Plt = platelets, Alb = albumin, T-Bil = total bilirubin, BUN = blood urea nitrogen, Cr = creatinine.

* *P* values were calculated using the unpaired *t-*test, the Mann-Whitney U test, and the χ^2^ test with Yates’ correction, as appropriate.

With the four factors (sex, hematocrit, albumin, and creatinine) that were selected in ascending order of *P* value, multivariable logistic regression analysis was performed. The multivariable logistic regression analysis using the four risk factors showed that a low albumin level was an independent risk factor for TSM (*P* = .035). Although male sex was predictive on univariable analysis (*P* = .049), it was not found to be an independent risk factor for TSM on multivariable analysis (*P* = .24). Although the values of hematocrit and creatinine were borderline predictors of TSM on univariable analysis, they were also not found to be independent risk factors for TSM on multivariable analysis (*P* = .98 and .12; Table 2).

**Table 2:** Factors related to arterial phase artifacts in the clinical study: Logistic regression analysis.

Characteristic	Odds ratio	*P* value
**Sex**	0.40 (0.08, 1.76)	.24
**Ht, %**	1.00 (0.88, 1.13)	.98
**Alb, g/dL**	4.46 (1.20, 20.6)	**.035**
**Cr, mg/dL**	0.086 (0.0029, 1.5)	.12

Note.—Numbers in parentheses are 95% confidence intervals.

Ht = hematocrit, Alb = albumin, Cr = creatinine.

### Results of the animal study

#### Gadoxetate disodium, PBS and gadopentetate dimeglumine administration into the distal IVC

**Fig 4:**
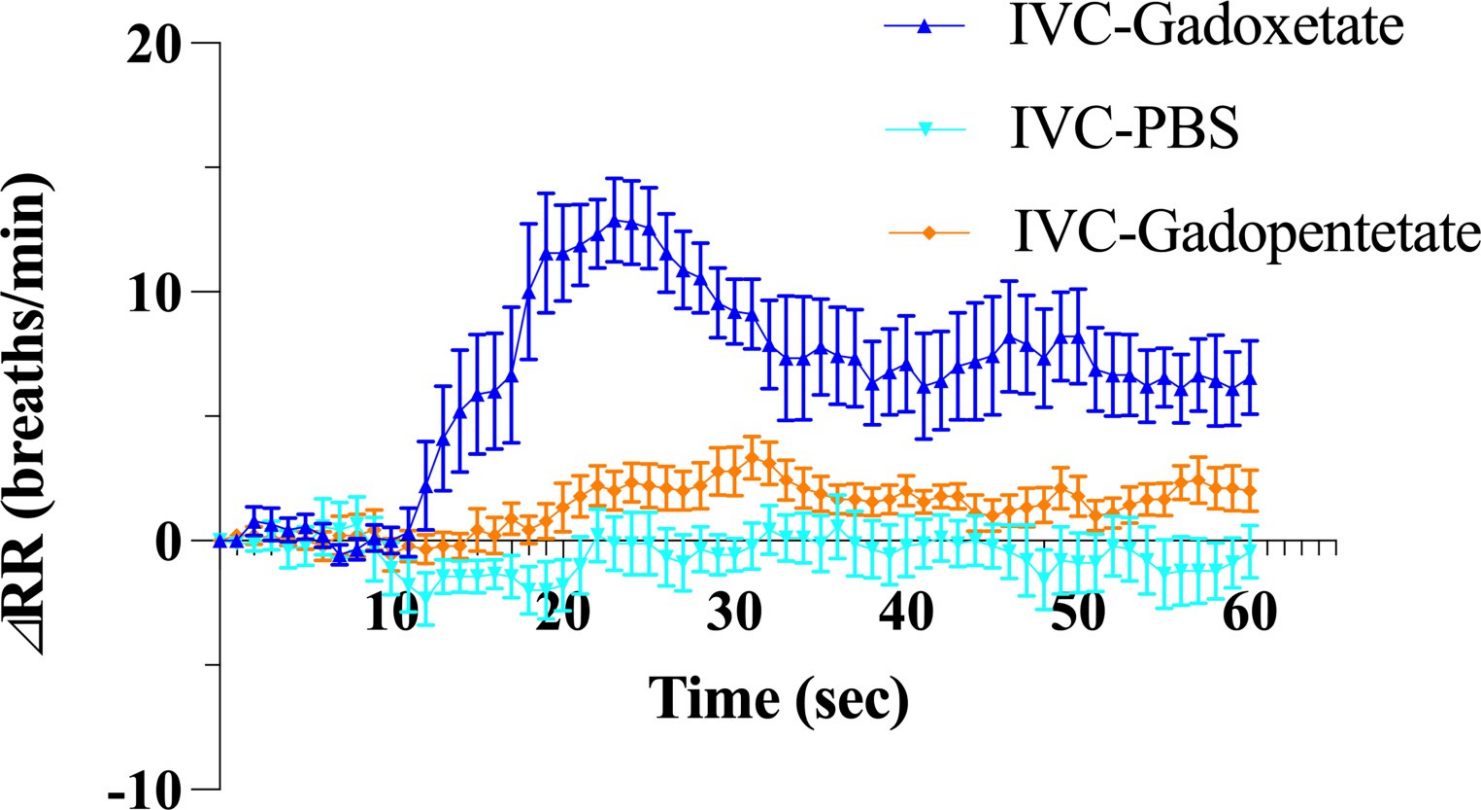
Differences in the time course of the respiratory rate among the different materials. Note.—Error bars represent standard errors (n = 9 rats per group). IVC-Gadoxetate, gadoxetate disodium injection into the distal inferior vena cava (IVC); IVC-PBS, phosphate-buffered saline injection into the distal IVC; IVC-Gadopentetate, gadopentetate dimeglumine injection into the distal IVC.

All 41 rats showed transient tachypnea with an elevation in RR of more than 8 breaths/min (approximately 15% increase compared to baseline) with gadoxetate disodium administration. On the other hand, with PBS and gadopentetate dimeglumine injection into the distal IVC, the maximum elevations in RR were significantly lower than gadoxetate disodium (Fig 4, Table 3).

**Table 3:** Statistical analysis of the maximum elevation in the respiratory rate among different materials.

	Gadoxetate	PBS	Gadopentetate	*P* value		
				Gadoxetate vs PBS	Gadoxetate vs Gadopentetate	PBS vs Gadopentetate
**Max ΔRR, breaths/min**	16.6 (1.5)	3.0 (0.8)	4.3 (0.7)	**< .001**	**< .001**	.66

Note.—The value of the maximum elevation in the respiratory rate after injection of phosphate-buffered saline (PBS) -diluted gadoxetate disodium, PBS and PBS-diluted gadopentetate dimeglumine are shown, and comparisons among different materials were evaluated using one-way analysis of variance and Holm-Sidak’s multiple comparisons test. All values are shown as means (standard error). Max ΔRR defined in Fig 3 was recorded for each individual rat, and the mean and standard error for each group were calculated. Thus, the values in this table may not necessarily appear to be the same as in Fig 4.

Gadoxetate = PBS-diluted gadoxetate disodium injection into the distal IVC, PBS = PBS injection into the distal IVC, Gadopentetate = PBS-diluted gadopentetate dimeglumine injection into the distal IVC, ΔRR = change (increase) in the respiratory rate compared to 0 sec.

#### DSA and gadoxetate disodium administration into the distal IVC, ascending aorta, and descending aorta

**Fig 5:**
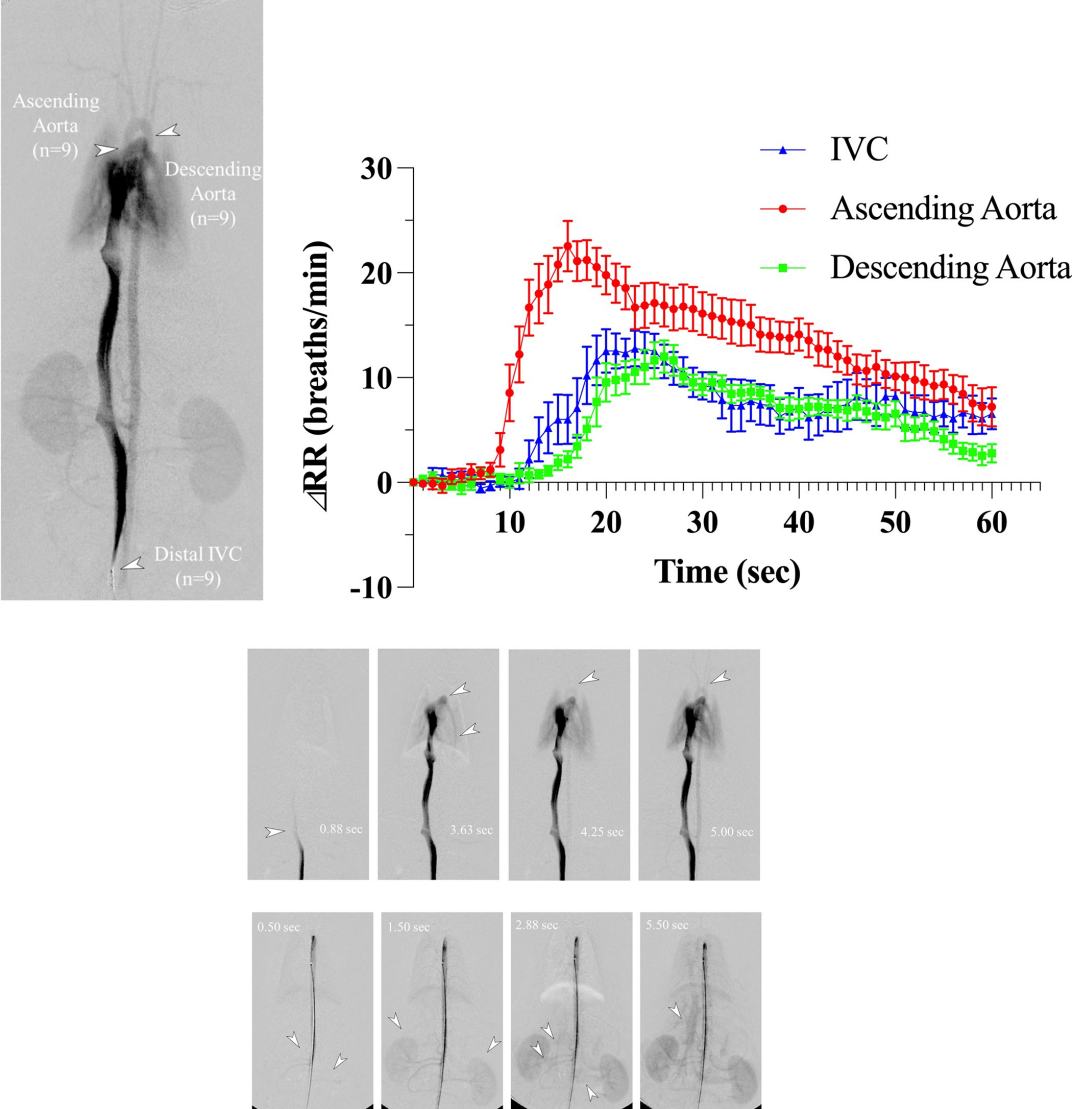
(a) Three injection sites are shown on digital subtraction angiography in rats. **(b)** Differences in the time course of the respiratory rate among the different injection sites. Note–Error bars represent standard errors (n = 9 rats per group). IVC, gadoxetate disodium injection into the distal inferior vena cava (IVC); Ascending aorta, gadoxetate disodium injection into the ascending aorta; Descending aorta, gadoxetate disodium injection into the descending aorta; ΔRR, change (increase) in the respiratory rate. **(c)** Representative digital subtraction angiography with distal IVC injection. The arrowhead shows the IVC at the level of the right renal vein at 0.88 sec, proximal and distal pulmonary artery at 3.63 sec, aortic arch branches at 4.25 sec, and aortic arch branches clearly at 5.00 sec. **(d)** Representative digital subtraction angiography with descending aorta injection. The arrowhead shows the bilateral renal arteries at 0.50 sec, bilateral kidneys at 1.50 sec, bilateral renal veins and IVC at 2.88 sec, and the IVC clearly at 5.50 sec.

27 rats were used to evaluate the relationship between the injection sites of the contrast agent and the time course of RR (Fig 5A). The time course of RR for the three injection sites is shown in Fig 5B and Table 4. With gadoxetate disodium injection into the distal IVC, the maximum elevation in RR was approximately 16.6 breaths/min at 18.4 sec after the start of injection, and tachypnea started at 15.6 sec. On the other hand, with injection into the ascending aorta, the maximum elevation in RR was approximately 24.6 breaths/min at 15.6 sec, and tachypnea started at about 10.3 sec. In addition, with injection into the descending aorta, the maximum elevation in RR was approximately 12.7 breaths/min at 22.7 sec, and tachypnea started at about 17.9 sec. The starting time of tachypnea was significantly earlier with injection into the ascending aorta than into the distal IVC or the descending aorta (both *P* < .001). Moreover, the starting time of tachypnea was significantly earlier with injection into the distal IVC than into the descending aorta (*P* = .001). The time of the maximum elevation in RR was significantly earlier with injection into the ascending aorta than with injection into the distal IVC or the descending aorta (*P* = .028 and < .001, respectively). The differences in the starting time of tachypnea between the ascending aorta and the others, the distal IVC and the descending aorta, were approximately 5.3 sec and 7.6 sec, respectively. It was 2.3 sec between the distal IVC and the descending aorta.

**Table 4:** Statistical analysis of time-series changes in the respiratory rate among different injection sites in rats.

Characteristic	IVC	A-Ao	D-Ao	*P* value		
				IVC vs A-Ao	IVC vs D-Ao	A-Ao vs D-Ao
**Starting time of tachypnea, sec**	15.6 (1.2)	10.3 (0.3)	17.9 (0.7)	**< .001**	.051	**< .001**
**Time of max ΔRR, sec**	18.4 (0.8)	15.6 (0.4)	22.7 (0.9)	**.028**	**.0014**	**< .001**
**Max ΔRR, breaths/min**	16.6 (1.5)	24.6 (2.7)	12.7 (1.5)	**.017**	.18	**< .001**

Note.—The starting time of tachypnea (the elevation in the respiratory rate more than 5 breaths/min compared to 0 sec) after injection of phosphate-buffered saline (PBS) -diluted gadoxetate disodium, the time and the value of the maximum elevation in the respiratory rate are shown, and comparisons among different sites of injection were evaluated using one-way analysis of variance and Holm-Sidak’s multiple comparisons test. All values are shown as means (standard error). These three parameters defined in Fig 3 were recorded for each individual rat, and the mean and standard error for each group were calculated. Thus, the values in this table may not necessarily appear to be the same as in Fig 5B.

IVC = PBS-diluted gadoxetate disodium injection into the distal inferior vena cava, A-Ao = PBS-diluted gadoxetate disodium injection into the ascending aorta, D-Ao = PBS-diluted gadoxetate disodium injection into the descending aorta, ΔRR = change (increase) in the respiratory rate compared to 0 sec.

The value of the maximum elevation in RR was significantly higher in the ascending aorta group than in the distal IVC group and in the descending aorta group, (*P* = .017 and < .001, respectively). As shown in the representative example, DSA with iodinated contrast agent injected into the distal IVC required approximately 0.88 sec to visualize the IVC at the level of the right renal vein, and 4.25 sec to visualize the ascending aorta and carotid arteries (Fig 5C). In addition, DSA with iodinated contrast agent injected into the descending aorta required approximately 2.88 sec to visualize the IVC at the same level (Fig 5D).

#### Gadoxetate disodium administration in rats: effect of dilution with 5% albumin instead of PBS

**Fig 6:**
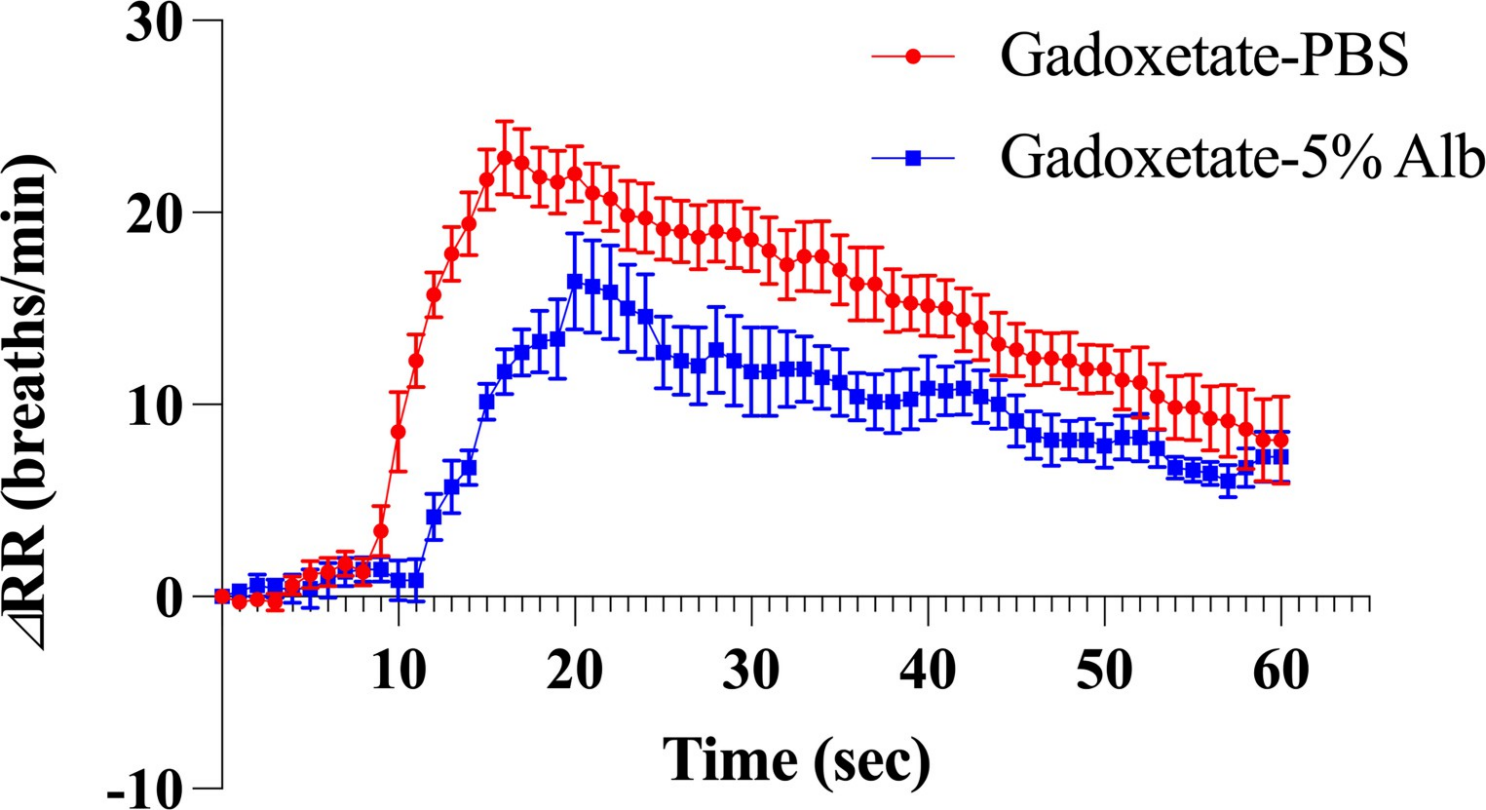
Differences between dilution of gadoxetate disodium with 5% serum albumin and with phosphate-buffered saline. Note.—Error bars represent standard errors (n = 7 rats per group). Injection of phosphate-buffered saline-diluted gadoxetate disodium into the ascending aorta shows the maximum elevation in the respiratory rate of 24.1 breaths/min at 15.7 sec from the start of injection, and tachypnea starts at 9.9 sec. On the other hand, injection of 5% albumin-diluted gadoxetate disodium shows the maximum elevation of 17.0 breaths/min at 18.1 sec from the start of injection, starting at 13.0 sec. Gadoxetate-PBS = injection of phosphate-buffered saline-diluted gadoxetate disodium; Gadoxetate-5% Alb = injection of 5% albumin-diluted gadoxetate disodium; ΔRR = change (increase) in the respiratory rate.

In addition, after injection of PBS-diluted gadoxetate disodium into the ascending aorta in 7 rats, the maximum elevation in RR was approximately 24.1 breaths/min at 15.7 sec, and tachypnea started at 9.9 sec from the start of injection (Fig 6 and Table 5). On the other hand, with 5% albumin-diluted gadoxetate disodium in 7 rats, the maximum elevation in RR was approximately 17.0 breaths/min at 18.1 sec, and tachypnea started at about 13.0 sec. With dilution with 5% albumin, the starting time was significantly delayed (*P* < .001), and the maximum elevation in RR was significantly delayed and suppressed (*P* = .020 and = .031, respectively).

**Table 5:** Statistical analysis of time-series changes in the respiratory rate between dilution methods.

Characteristic	Gadoxetate-PBS	Gadoxetate-5% Alb	*P* value
**Starting time of tachypnea, sec**	9.9 (0.3)	13.0 (0.4)	**< .001**
**Time of max ΔRR, sec**	15.7 (0.4)	18.1 (0.8)	**.020**
**Max ΔRR, breaths/min**	24.1 (1.8)	17.0 (2.3)	**.031**

Note.—The starting time of tachypnea (the elevation in the respiratory rate more than 5 breaths/min compared to 0 sec) after injection of contrast agents into the ascending aorta, the time and the value of the maximum elevation in the respiratory rate are shown. Dilution methods were compared using the unpaired *t*-test. All values are shown as means (standard error). These three parameters defined in Fig 3 were recorded for each individual rat, and the mean and standard error for each group were calculated. Thus, the values in this table may not necessarily appear to be the same as in Fig 6.

Gadoxetate-PBS = phosphate-buffered saline (PBS) -diluted gadoxetate disodium injection into the ascending aorta, Gadoxetate-5% Alb = 5% albumin-diluted gadoxetate disodium injection into the ascending aorta, ΔRR = change (increase) in the respiratory rate compared to 0 sec.

## Discussion

Various studies of transient motion artifacts after administration of contrast agents have been published [15–19]. However, the cause remains to be clarified. The goal of the present study was to identify risk factors for TSM on free-breathing MRI in a clinical study, and investigate the cause and the organ which triggers respiratory irregularities in an animal study. In the retrospective clinical study, a multivariable analysis identified a low albumin level as a risk factor for TSM. To the best of our knowledge, this has not been previously reported, since serum albumin was not evaluated in previous studies. In previous studies with breath-holding MRI, chronic obstructive pulmonary disease, high body mass index, and male sex were reported as risk factors for TSM [14, 20]. Chronic obstructive pulmonary disease was not significant in the present study, possibly because of its low prevalence in this cohort, or the effect of impaired breath-holding fidelity may have been attenuated under free breathing. Male sex was a risk factor on univariable analysis, but not on multivariable analysis, and high body mass index was not identified as a risk factor, possibly due to the small sample size.

### Breath holding may suppress respiratory irregularities

The reported rate of severe motion artifacts during breath holding is 8–20% [8, 10, 13–15, 17, 21, 22]. On the other hand, the rate was 39.2% in our study with free breathing. Although the definition was different, a prospective observational cohort study reported that the rate of transient respiratory motion during free breathing was 70.7%, indicating a higher rate than breath holding [12]. In addition, it is reported that TSM could not suppressed by intensified pre-scan patient preparation [23]. Moreover, similar to a previous report in mice, all rats showed tachypnea with gadoxetate disodium administration under free breathing in our animal study [18]. These paradoxical states can be explained by the hypothesis that respiratory irregularities may originally occur unconsciously, e.g. not a failure in breath holding, but the movement of the abdominal wall could be suppressed by breath holding. Thus, to some extent, TSM may be suppressed with breath holding.

### Gadoxetate disodium, gadopentetate dimegumine and PBS administration into the distal IVC and differences in injection sites

In our animal study in rats, gadoxetate disodium administration into the distal IVC caused significant elevation in RR compared to gadopentetate dimeglumine or PBS.

Injection of gadoxetate disodium into the ascending aorta caused tachypnea earlier than into the distal IVC by 5.3 sec and the descending aorta by 7.6 sec. This time gap with the distal IVC was similar to the time needed to visualize the carotid artery on DSA from the distal IVC (approximately 4.25 sec). Thus, we consider that tachypnea may have occurred earlier with injection into the ascending aorta because gadoxetate disodium reached the aortic arch branches earlier. Similarly, to visualize the IVC at the level of the right renal vein, it was approximately 2.0 sec earlier with injection into the distal IVC than into the descending aorta, and the difference of their starting time of tachypnea was 2.3 sec. From these results, we speculate that TSM is triggered by the effect of gadoxetate disodium on the head and neck region, such as the respiratory center or carotid body, rather than the chest or abdominal region. However, further investigation is needed to identify the exact organ. The high value of the maximum elevation in RR in the ascending aorta group is thought to be related to the high concentration of gadoxetate disodium at aortic arch branches, since it was previously reported that dilution of gadoxetate disodium suppressed the artifacts [24].

### Effect of serum albumin on respiratory irregularities

Dilution of gadoxetate disodium with 5% serum albumin instead of PBS delayed and suppressed tachypnea after injection into the ascending aorta. This result supports the result of the clinical study showing that serum albumin is related to gadoxetate disodium-induced TSM.

Gadoxetate disodium is a contrast agent that has a lipid-soluble side chain, an ethoxybenzyl group, bound to a gadopentetate molecule [1]. Generally, lipophilic drugs are more likely to permeate into tissue or be bound to serum albumin, which determines the ease of transfer into tissues [25–28]. In fact, gadoxetate disodium has a larger volume of distribution and a higher serum protein binding ratio than gadopentetate dimeglumine [1]. Thus, gadoxetate disodium is more likely to be unbound with a low albumin level. In addition, with hypoalbuminemia, the effect of some lipophilic drugs is known to become stronger because more drug molecules become unbound, supporting the hypothesis that the pharmacological effect of gadoxetate disodium caused TSM [29]. Therefore, we consider that the pharmacological effect of unbound gadoxetate disodium would be stronger and highly related to the occurrence of TSM with a low albumin level. Moreover, a dose-dependent occurrence of respiratory motion with gadoxetate disodium has been reported [25, 30], which corroborates our hypothesis, because more unbound molecules will exist. After injection into the ascending aorta, the solution immediately reached the carotid arteries. However, dilution with 5% serum albumin clearly delayed and suppressed tachypnea even though we started injection at the same time. We speculate that this is due to the binding of gadoxetate disodium to serum albumin molecules and the reduction of unbound gadoxetate disodium.

The present study has some limitations. First, the number of patients in this single-center retrospective clinical study was small. Though serum albumin was the focus of the study, further investigation is required to confirm the result obtained. A further limitation of this study is that our animal study did not replicate gadoxetate disodium administration in patients with hypoalbuminemia. By establishing two groups with different albumin levels, the effect of albumin will be further clarified. In addition, using the tail artery and vein would be less invasive. Moreover, we could not identify the exact organ which triggers TSM, though it was related to the effect of gadoxetate disodium on the head and neck region. However, although there are some limitations, the effect of serum albumin on respiratory irregularities was obvious, at least in anesthetized rats. In addition, definitions of respiratory irregularities and image interference were simplified to focus on causes. Factors that contribute to the original degree of irregularities may have been hidden. Image interpretation, albeit simplified, was inadvertently biased because blind readers are always operator-dependent.

In conclusion, a low albumin level might be a risk factor for TSM in patients, and it was confirmed that serum albumin suppresses respiratory irregularities in rats in our experimental model. Furthermore, the present data suggest that the respiratory irregularities could be caused by the effect of gadoxetate disodium on the head and neck region rather than on the chest or abdominal region.

## Supporting information

S1 File (XLSX)

## Abbreviations

TSM: transient severe motion
RR: respiratory rate
IVC: inferior vena cava
DSA: digital subtraction angiography
PBS: phosphate-buffered saline
